# 抗氧化治疗在放射性肺损伤中的研究进展

**DOI:** 10.3779/j.issn.1009-3419.2019.09.05

**Published:** 2019-09-20

**Authors:** 营歌 李, 启斌 宋, 颐 姚, 熠 董, 彦君 高, 彬 吴

**Affiliations:** 430060 武汉，武汉大学人民医院肿瘤中心 Cancer Center, Renmin Hospital of Wuhan University, Wuhan 430060, China

**Keywords:** 活性氧, 抗氧化治疗, 放射性肺损伤, 放疗防护, Reactive oxygen species, Anti-oxygen therapy, Radiation-induced lung injury, Radiation protection

## Abstract

放射性肺损伤（radiation induced lung injury, RILI）是临床上胸部肿瘤患者放疗后发生的严重并发症，主要表现为气短、低热、咳嗽等，严重影响患者生存，如何更好地防治RILI是亟待探索的问题。RILI的发生机制主要包括靶细胞学说、细胞因子学说、自由基学说、血管内皮细胞损伤学说。其中放疗产生的活性氧（reactive oxygen species, ROS）对组织的损伤贯穿整个RILI病程，对放射性肺炎和放射性肺纤维化均具有直接促进作用。包括巯基化合物、抗氧化酶、植物抗氧化剂等在内的治疗手段已被用于防治RILI，本文即就抗氧化治疗在RILI中的研究与应用作一综述。

## Abstract (en)

Radiation induced lung injury (RILI) is a serious complication in patients received thoracic radiotherapy. The main clinical symptom of RILI includes short of breath, low fever and cough, seriously a?ect the survival of patients. How to beter prevent and treat RILI is an urgent problem. Target theory, cytokine theory, free radical theory, and vascular endothelial cell damage theory are the main mechanisms of RILI. Among them, reactive oxygen species (ROS) produced during radiotherapy can induce tissue damage throughout the course of RILI, and have a direct effect on both radiation pneumonitis and radiation-induced lung fbrosis. Anti-oxygen therapy including thiol compounds, antioxidant enzymes, and plant antioxidants have been applied in the prevention and treatment of RILI. This article reviews the research and application of antioxidant therapy in RILI.

放射治疗被认为是肿瘤治疗最重要的基本手段之一，尽管放疗技术日益精进，其带来的不良反应仍不可避免。而肺组织对放疗的毒性反应敏感，成为放射治疗最主要的剂量限制器官^[1]^。放射性肺损伤（radiation induced lung injury, RILI）是胸部放疗后常见的并发症。氧化应激在RILI发生中起着至关重要作用。随着人们对RILI分子机制和病理机制的逐步阐明，抗氧化治疗对RILI的意义日益重要。本文将就抗氧化治疗在RILI中的研究进展作一综述。

## 1 放射性肺损伤概述

RILI是胸部肿瘤（肺癌、食管癌、乳腺癌、恶性淋巴瘤等）患者接受放疗后发生的，因肺组织受照射而引起的炎症反应，一般发生在放疗后1个月-3个月，早期表现为放射性肺炎（radiation pneumonitis, RP），晚期表现为放射性肺纤维化（radiation induced lung fibrosis, RILF），二者界限不确定，常波动于数周至6个月不等^[2]^。RILI发生同正常肺组织受照射体积呈正相关，研究表明，接受20Gy及以上照射剂量的正常肺组织的体积（V_20_）为30%-40%时，急性RP发生率为13%，V_20_超过40%，急性RP的发生率则高达36%^[3]^，因此正常肺组织限量至关重要，目前被广泛接受的剂量限制方案包括：三维适形放疗:全肺平均剂量（Mean Lung Dose, MLD）≤20Gy, V_20_≤37%（RTOG0617）; V_20_≤35%（RTOG0972/CALGB36050）; 立体定向体部放疗: V_20_≤10%（RTOG0618，3次）; V_20_＜5%-10%（ROSEL European trial，3次或5次）^[4]^。RILI的发生机制主要包括传统的靶细胞学说、细胞因子学说、自由基学说、血管内皮细胞损伤学说等^[5-7]^。RILI的始动因素一方面来自于电离辐射能量直接作用于DNA，另一方面来自于水分子受激发和电离后产生大量活性氧（reactive oxygen species, ROS）间接作用导致蛋白质、核酸等生物大分子的氧化损伤，直接影响一系列基因的转录与表达，最终导致细胞损伤或死亡^[8-9]^。后者的损伤潜能或许更大，不仅作用于照射早期，还可持续作用至纤维化形成阶段^[10]^; 不但引起照射部位损伤，还可引起非照射部位损伤，如旁观者效应（bystander effect），早期旁观者效应的发生主要是由线粒体依赖的ROS增加引发的，首先是引发复杂的细胞间信号传导，进一步持续导致细胞DNA的损伤^[11]^。因此氧化应激在RILI发生中具有重要作用，值得深入探索。

## 2 活性氧与RILI

ROS是指一类氧化性较强的未被完全还原的氧分子，来源于水分子的电离辐射和照射后细胞的线粒体呼吸链，包括羟基自由基（•OH），氢过氧自由基（HOO•），过氧化氢（H_2_O_2_）和超氧阴离子（•O_2^-^_）等，通过调节多个细胞信号传导通路（如NF-κB和STAT3），以及缺氧诱导因子1α（hypoxia-inducible factor 1α, HIF-1α）和多种激酶、生长因子、细胞因子等，参与细胞转化、炎性浸润、组织损伤甚至诱发恶变^[12]^。

ROS主要通过诱导白细胞介素-1（interleukin-1, IL-1）、IL-4、IL-6、IL-13、肿瘤坏死因子α（tumor necrosis factor-α, TNF-α）、干扰素γ（interferon-γ, IFN-γ）等从受照射组织中释放参与RP的发生^[13]^。与此同时，ROS还可参与免疫细胞的激活，主要机制为ROS负性调控蛋白酪氨酸磷酸酶活性，作为第二信使启动并放大下游淋巴细胞活化信号，导致淋巴细胞活化^[14, 15]^。在组织水平，局部ROS堆积及氧化应激导致血管内皮细胞损伤及功能障碍，内皮细胞间连接被破坏，炎性细胞穿过内皮屏障，参与组织的炎症反应^[16]^。急性期反应后，ROS在纤维化进程中同样重要。肌成纤维细胞在RILF中占据主导地位，其来源主要为正常成纤维细胞的放射活化、肺泡上皮细胞的上皮间质转化（epithelial-mesenchymal transition, EMT）等^[17]^。而ROS则被证明可以促进肺泡上皮细胞的EMT和肺成纤维细胞的纤维化表型的形成^[18-19]^、促进成纤维细胞的活化及胶原的产生^[20]^。因此，抗ROS治疗可能是防范RILI发生的关键。

## 3 抗氧化治疗

目前在研的抗氧化剂主要包括巯基化合物、抗氧化酶及类似物、植物抗氧化剂等^[21-22]^。

### 3.1 巯基化合物

巯基化合物主要包括阿米福汀（amifostine, WR272）、还原性性谷胱甘肽（glutathione, GSH）及其前体N-乙酰半胱氨酸（N-acetylcysteine, NAC）等，主要作用机制是利用其侧链的活泼巯基与自由基结合，中和自由基以保护自身蛋白上的巯基不被氧化，其中GSH还可以在谷胱甘肽过氧化物酶的作用下与H_2_O_2_结合将其还原为H_2_O。Devine等^[23]^比较了16项临床研究中，阿米福汀对1057例接受放化疗或单独放疗的非小细胞肺癌（non-small cell lung cancer, NSCLC）的放疗毒性和疗效的影响，结果发现阿米福汀可以使急性肺毒性的发生风险降低44%（RR: 0.56; 95%CI: 0.41-0.75, *P*=0.000, 1），且不影响治疗效果。瞿述根等^[24]^研究了GSH腹腔注射分别对接受低剂量（15 Gy）和高剂量（30 Gy）照射小鼠的影响，结果发现相较于单纯照射组，GSH的应用可有效提高脾脏指数，降低血清中基质金属蛋白酶、IL-6的水平，对RILI具有保护作用，其中低剂量照射组更明显。在GSH用于RILI的临床探索中，杨新华等^[25]^将174例接受放疗的NSCLC患者随机分为两组，实验组放疗的同时给予GSH 2.4 g/d，对照组单纯放疗。结果提示有效率：治疗组为89.7%，对照组为92.0%;而RP和RILF的发生率：治疗组为9.2%和36.8%，对照组为21.8%和54.0%，差异均有统计学意义（*P*＜0.05）。王玉娟等^[26]^的研究结果则表明GSH不但能够降低局部晚期肺癌患者RP和RILF的发生率（RP从46.5%降至20.9%，RILF从27.9%降至11.6%），还提高了放疗疗效，其中GSH组近期总有效率（76.7%）明显高于对照组（51.2%），差异均有统计学意义（*P*＜0.05）。NAC是GSH的前体物质，Han等^[27]^开展的前瞻性研究则证明雾化应用NAC可以减少RP患者的痰液产生，减少祛痰剂的使用。以上研究证实，无论是阿米福汀还是GSH，均可以在不影响甚至提高放疗疗效的前提下改善肺组织炎症反应，减少RILI的发生。而NAC虽然被证实有利于RILI症状的缓解，但尚缺乏其遏制RILI的直接证据。

### 3.2 抗氧化酶

为维护自身的氧化还原平衡，对抗ROS，机体还存在一套抗氧化酶体系，包括超氧化物歧化酶（superoxide dismutase, SOD）、谷胱甘肽过氧化物酶（glutathione peroxidase, GPx）、过氧化氢酶等，其中SOD可以将2分子的•O_2^-^_还原为H_2_O_2_和O_2_，而H_2_O_2_则进一步被过氧化氢酶和GPx还原为H_2_O。目前在RILI防治领域关于SOD的研究较多，Murigi等^[28]^在给小鼠全肺照射后，给予SOD小分子类似物（AEOL 10150）25 mg/kg/d治疗，共28天，结果相较于单纯照射组，AEOL 10150治疗组肺水肿和肺充血显著减少（*P*＜0.02），小鼠存活率也显著改善。Antonic等^[29]^发现单侧肺部放疗导致大鼠呼吸频率增加、组织病理学改变、氧化应激、巨噬细胞活化和转化生长因子β（transforming growth factor β, TGF-β）的表达增加，而放疗后24 h皮下注射15 mg/kg牛SOD可明显改善上述参数，因而证实单次应用SOD（15 mg/kg）可通过抑制ROS物质及活性氮物质的产生进而改善RILI。

### 3.3 植物抗氧化剂

自然界中有大量植物天然含有抗氧化成分。近年来，植物抗氧化剂因其来源广、低毒、病人耐受性好等优势，在放射防护领域逐渐获得重视。橙皮苷（hesperidin）是来自于芸香科柑橘类物种的二氢黄酮类化合物，具有抗氧化性能。研究发现，与单纯放疗组相比，照射前连续7 d应用橙皮苷（100 mg/kg）可明显改善大鼠放疗后8周肺组织炎症反应、纤维化和肥大细胞浸润（*P*＜0.05）^[30]^。同样作为柑橘类果皮或种子中的提取物，柚皮素也可作为RILI的另一种潜在治疗药物。Zhang等^[31]^发现，柚皮素可以通过降低IL-1β水平及调节炎症因子水平减少小鼠肺部照射后炎症的产生。藜芦醇苷（polydatin）是一种植物多酚类药物，因其抗氧化性被用于RILI防护的探索，Cao等^[32]^研究证实白藜芦醇苷可以抑制TGF-β1/Smad3信号通路和EMT，还可以缓解辐射诱导的Th1/Th2失衡，显示了潜在放射防护作用。

## 4 展望

尽管抗氧化剂在RILI防护中显示出了巨大潜力，然而要实现临床应用还需关注以下几方面问题：首先，抗氧化剂作为正常组织放疗保护剂，同时是否也是肿瘤细胞的保护伞？尚需进一步研究。其次，RILI的发生涉及一系列细胞、分子、组织病理的复杂动态变化，且显示出个体差异^[33]^，究其原因是否与某些基因的表达相关？将来应用于临床时，患者是否需要经过筛选？第三，ROS的产生可能随放疗的体积、剂量、分割、疗程、同期治疗的类型变化而变化，要达到真正的放射防护，抗氧化剂的应用时机、疗程就应当随之变化，因此，当中的个体化、规范化问题还有待进一步探索。最后，大部分抗氧化剂的研究仅停留在动物试验阶段，尚需更规范和大规模的临床研究证据支持，不仅需要涉及考察抗氧化剂对RILI的防范作用，还要关注药物同时对放疗疗效的影响。
